# The ‘Influenza’ Vaccine Used during the Samoan Pandemic of 1918

**DOI:** 10.3390/tropicalmed3010017

**Published:** 2018-02-02

**Authors:** G. Dennis Shanks

**Affiliations:** 1 Australian Defence Force Malaria and Infectious Disease Institute, Enoggera, QLD 4051, Australia; dennis.shanks@defence.gov.au; Tel.: +61-7-3332-4931; Fax: +61-7-3332-4800; 2 University of Queensland, School of Public Health, Herston, QLD 4006, Australia

**Keywords:** influenza, 1918 pandemic, vaccine, endotoxin

## Abstract

In 1918, a crude influenza vaccine made from chemically inactivated, mixed cultures of respiratory bacteria was widely used prior to the understanding that influenza was caused by a virus. Such vaccines contained no viral material and probably consisted largely of bacterial endotoxin. The Australian military used such a vaccine on Samoa in December 1918 and thought it was valuable. Post hoc analyses suggest that the mixed respiratory bacteria vaccine may have actually been of some benefit, but the mechanism of such protection is unknown. Although such a crude vaccine would not be considered in a modern setting, the rapid use of problematic vaccines still remains a risk when new influenza types suddenly appear, as in 1976 and 2009.

Epidemic lethal infections hold a particular terror for humans that has only been partially resolved by the modern medical practice of immunization. When unfamiliar infections appear, there is a rush to prepare protective vaccines, as seen during the 2015 Ebola epidemic in West Africa. These efforts go back over the previous century, when our medical understanding of immunity was only beginning to be formed. Ever since Louis Pasteur demonstrated that vaccination protected people against the otherwise universally lethal rabies virus, there has been a desire to counter exotic lethal infections with immunizations. The influenza pandemic of 1918–1920 arrived at the end of the First World War, and although the exact number of deaths caused by influenza will never be known, the global toll clearly exceeded those killed in the war by several fold [1]. Australia had warning of the pandemic from Europe, which gave the Commonwealth Serum Laboratories (a predecessor of the current CSL) time to prepare a vaccine. Since *Haemophilus influenzae* was thought to be the causative agent from the previous 1890 pandemic, a mixture of common respiratory bacteria was made into a vaccine and given to more than 400,000 Australians [2]. This vaccine predated our understanding of the existence of viruses; the influenza virus itself was not isolated until the 1930s [3]. Although no one would make or use such a vaccine today, it was all that was available when the Pacific island state of Samoa was devastated by influenza. As it was the primary intervention used by the Australian military medical relief mission sent to Samoa in December 1918, it is illustrative to examine how this vaccine was employed [4].

The tragic narrative of how the SS *Talune* (Figure 1A) brought influenza and thus mass mortality from Auckland, New Zealand to Fiji, Tonga, and Samoa with a ship captain, who denied knowing that influenza was an infectious disease, has been previously described [5,6,7,8,9].

News of the end of the First World War arrived just as Samoans began to die of influenza. By mid-November, it was clear that a disaster was underway and an urgent call for help went from Apia, Samoa to Wellington, New Zealand [6]. As New Zealand was also facing pandemic influenza, the as-yet-unaffected Australia was asked to send help in the form of a medical relief team on the naval cruiser HMAS *Encounter* (Figure 1B) [10]. Loaded in record time in Sydney with a refueling stop in Suva, Fiji, HMAS *Encounter* made rapid progress towards the stricken Samoan islands. The local European community mobilized to assist the Samoan population with food and water delivery to homes, a critical issue when everyone in a house became simultaneously ill [5]. HMAS *Encounter* arrived off Apia, Samoa on 3 December 1918 as the influenza epidemic was waning and assisted with the on-going medical relief. The ship’s crew, along with the local garrison of the New Zealand Army, managed the difficult task of burying thousands of bodies as nearly the entire local population was incapacitated (Figure 2) [5,6,11]. Immunization with the ‘mixed bacterial’ vaccine proceeded even though most Samoans by that time had already been infected. The estimated number of Samoan deaths of between 8000 and 9000 is similar to that in all of New Zealand and not far short of the 11,000 dead in Australia, countries with 25 and 125 times Samoa’s population, respectively [7,8,9,12].

The Commonwealth Serum Laboratories in Australia produced a special vaccine mixture of chemically killed bacteria as a mélange of *Haemophilus influenzae, Streptococcus pneumoniae, Streptococcus pyogenes*, and *Neisseria* species (Figure 3) [4,5,13,14,15,16]. This vaccine was effectively a large injection of endotoxin-like material and other toll-like receptor (TLR) agonists, which was repeatedly given to the 450 crew members of HMAS *Encounter* [10,17]. HMAS *Encounter* had previously experienced influenza while the ship was off Western Australia in October 1918, when 16% of the sailors developed an influenza-like illness without any serious consequences. Once alerted to the need for a medical relief mission to Samoa at the end of November, sailors were given three to four sequential mixed bacterial vaccine injections such that they arrived off Samoa in early December ‘vaccinated’. While in Samoa, 0.4% of the ship’s crew developed influenza, although admittedly it was at the end of the epidemic [4,13,18]. When the influenza epidemic struck Australia in March 1919, 22% of the crew became ill, but none developed pneumonia or died [12]. No actual figures are available regarding the vaccine’s effectiveness in the Samoan community. This is far from the ‘miraculous’ result claimed by the senior naval surgeon on HMAS *Encounter*, but as in other ad hoc tests of mixed bacterial vaccine, the crude vaccine may have had a protective effect [12,13]. Bureaucratic arguments between the Governments of Australia and New Zealand continued for years concerning whose responsibility it was to pay for the relief mission and specifically the vaccines provided by the Australians to the people of Samoa [5].

Such a vaccine could do nothing specifically against the influenza virus, which had yet to be discovered. It is also difficult to see how such a mixed vaccine could generate an antibacterial immune response or how any such response would be matched to the particular serotype of the secondarily infecting organism(s) [19,20,21]. However, such crude vaccines had already been widely used in both England and the U.S., particularly in military populations [22,23,24,25]. These ‘mixed bacterial’ vaccines did seem to have some positive effect in preventing mortality, although this was judged in comparative military studies of questionable design that greatly pre-dated any understanding of controlled clinical trials [4,24,26,27]. Nevertheless, extensive analysis at the time and later using a more modern understanding of influenza seemed to confirm that at least some of the vaccines had a protective effect [28].

One speculative possibility is that what was effectively a large injection of adjuvant modified the clinical course of influenza. Could such a vaccine have caused a positive rebalancing of the immune system [29,30,31,32]? Pre-infection TLR stimulation protected mice against highly lethal influenza challenges [33]. Where influenza vaccine was followed by lymphocytic choriomeningitis virus (LCM) infection in mice, the resulting cross-reaction and severe immunopathology could be largely prevented by anti-interferon or peptide-toleration therapy [34]. When devising means to counter ‘original antigenic sin’ (influenza strains back-boosting previous exposures) by using sequential H1N1 infections/immunizations, investigators found that innate immune activators with TLR activity (*Pertussis* toxin, CpG, oligo-deoxynucleotides) effectively protected mice against lethal challenge. This occurred when the TLR agonist was given at the same time as the challenge virus by enhancing neutralizing antibodies [35]. There are multiple reasons to see influenza infections as a balance between host protection and immunopathology. Perhaps future interventions will attempt to move the equation in favor of the host [30,31,32,36,37].

Samoa in 1918 is only one example of when problematic vaccines were used during influenza pandemics. The 1976 National Influenza Immunization Program in the U.S. was a direct response to an epidemic in U.S. Army military trainees at Fort Dix, New Jersey of a strain of influenza virus thought to be similar to the 1918 pandemic virus. The public health decision to use an inactivated influenza vaccine based on the New Jersey strain and its political ramifications have been dissected ever since then. Although based on very competent public health advice, no subsequent pandemic appeared in 1976. Also, the occurrence of a few cases of a severe neurological disease, Guillain-Barré syndrome, meant that the entire immunization program was interpreted by the general public as a costly failure driven by political and not medical forces. Influenza immunization was unjustly discredited for a generation thereafter despite having delivered the specified vaccine on time.

Despite a better understanding of influenza pandemics and improved vaccines, when an H1N1 influenza virus that was very closely related to the 1918 strain did appear in 2009, it was still not possible to deliver a successful immunization program before the pandemic peaked in most populations. Despite improvements in vaccine technology, the requirement to clinically test any new product places an irreducible minimum time span on the interval from pandemic discovery to delivery of a pandemic vaccine [38]. Panic demand for a vaccine that still had to be prepared in hens’ eggs could not be initially satisfied due to the long lead time required to produce, test, approve, and field a new influenza vaccine. The virus strain chosen grew slowly in eggs, so the 2009 influenza pandemic in the U.S. was largely over by the time the new vaccine was ready. The panic caused by a new viral pandemic had largely disappeared once it became apparent that the 2009 influenza was not highly lethal, as was first suspected from initial reports from Mexico. Uptake of the 2009 vaccine was low, and the public impression was that the public health system had failed to deliver adequate protection against influenza despite the rapid production of an appropriate vaccine.

Current seasonal influenza vaccines are less than perfect (efficacy usually in the 60–80% range), but generally are well worth having when the viral strains chosen for the vaccine are well-matched to the circulating influenza viruses. Our interventions against infectious diseases will have to evolve along with the highly mutable influenza virus if we are to avoid having to use problematic vaccines again, such as that used in Samoa in 1918.

## Figures and Tables

**Figure 1:**
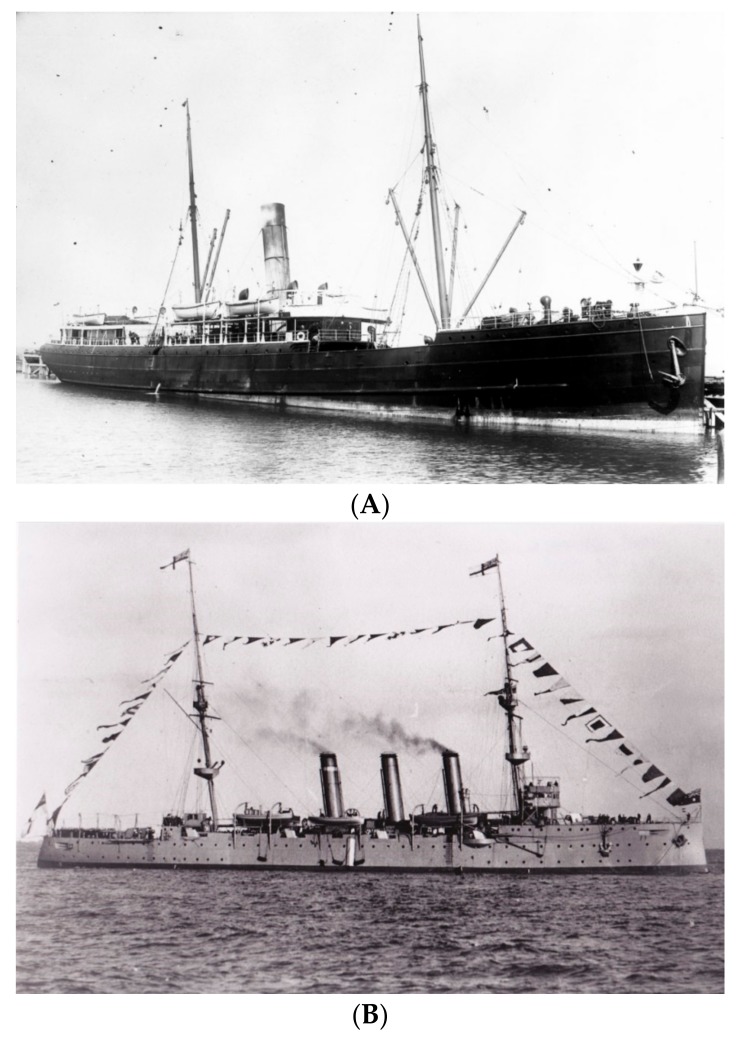
(**A**) The *SS Talune* shown in Napier, New Zealand in 1908, photo from https://nzhistory.govt.nz/media/photo/influenza-pandemic-hits-samoa, (Ministry for Culture and Heritage), updated 24 October 2014 and (**B**) The *Challenger* class light cruiser HMAS *Encounter* shown in Sydney, October 1913. Photo from http://www.navy.gov.au/hmas-encounter-i.

**Figure 2:**
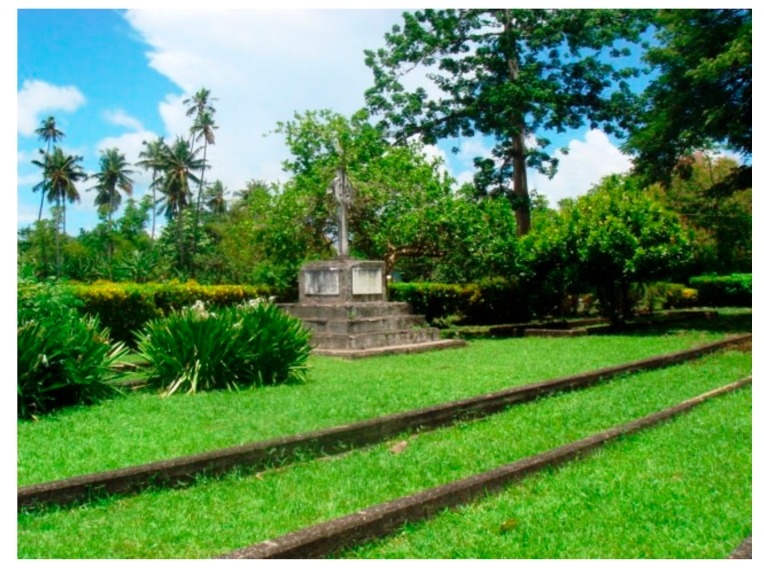
Mass grave of victims of the 1918 influenza pandemic in Apia, Samoa. Photo by Dr. Kevin Palmer.

**Figure 3:**
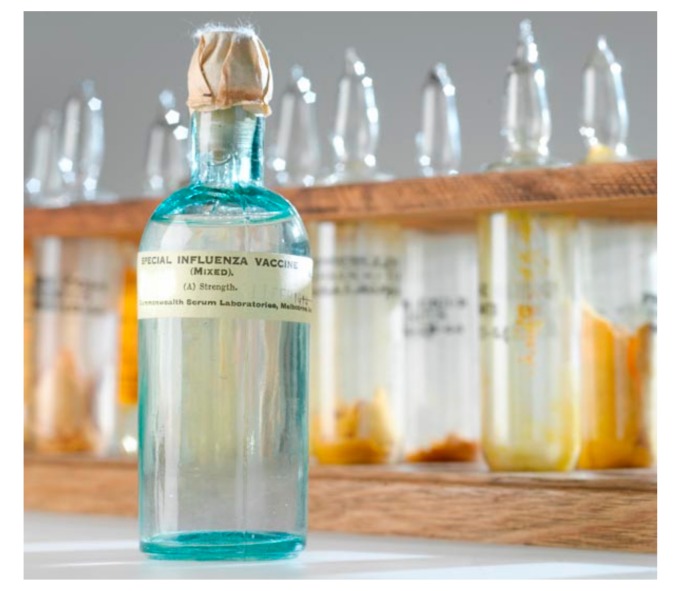
‘Special Influenza Vaccine’ as prepared by the Commonwealth Serum Laboratories in Australia in 1918 and used for the crew of HMAS *Encounter* during operations in Samoa. Photo by Michelle McFarland and copyright held by Museums Victoria as item 1230885.

## References

[1] Morens D.M., Taubenberger J.K., Fauci A.S. (2009). The persistent legacy of the 1918 influenza virus. N. Engl. J. Med..

[2] Curson P. (2015). Deadly Encounters: How Infectious Disease Helped Shape Australia.

[3] Beveridge W.I.B. (1977). Influenza: The Last Great Plague: An Unfinished Story of Discovery.

[4] Grey F. (1919). Influenza in Samoa: Value of vaccines. BMJ.

[5] Elliot G., Wilson T., Moorhouse W. (1919). Report of the Samoan Epidemic Commission.

[6] Tomkins S.M. (1992). The influenza epidemic of 1918–1919 in Western Samoa. J. Pac. His..

[7] Crosby A.W. (2003). America’s Forgotten Pandemic.

[8] Rice G.W. (2005). Black November: the 1918 Influenza Pandemic in New Zealand.

[9] McLane J.R. (2012). Setting a Barricade against the East Wind: Western Polynesia and the 1918 Influenza Pandemic. Ph.D. Thesis.

[10] Stevens D. The RAN and the 1918–1919 Influenza Pandemic, 2006. http://www.navy.gov.au/history/feature-histories/ran-and-1918-19-influenza-pandemic.

[11] Boyd M. (1968). The military administration of Western Samoa, 1914–1919. NZ. J. Hist..

[12] Cumpston J.H.L., Mullett J.A. (1919). Influenza and Maritime Quarantine in Australia.

[13] Grey F. (1919). Compulsory inoculation against Spanish influenza. Lancet.

[14] Shanks G.D., Brundage J.F. (2012). Pathogenic responses among young adults during the 1918 influenza pandemic. Emerg. Infect. Dis..

[15] Shanks G.D., Hussell T., Brundage J.F. (2012). Epidemiological isolation causing variable mortality in island populations during the 1918–1920 influenza pandemic. Influenza Other Respi Viruses.

[16] Shanks G.D., Brundage J.F. (2013). Pacific islands which escaped the 1918–1919 influenza pandemic and their subsequent mortality experiences. Epidemiol. Infect..

[17] Shanks G.D., Waller M., Smallman-Raynor M. (2013). Spatiotemporal patterns of pandemic influenza-related deaths in Allied naval forces during 1918. Epidemiol. Infect..

[18] Grey F. (1919). Notes on epidemic broncho-pneumonia (Spanish influenza) in Samoa. Med. J. Aust..

[19] Brundage J.F., Shanks G. (2008). Deaths from bacterial pneumonia during the 1918–1919 influenza pandemic. Emerg. Infect. Dis..

[20] Shanks G., MacKenzie A., McLaughlin R., Waller M., Dennis P., Lee S-E., Brundage J.F. (2010). Mortality risk factors during the 1918–1919 influenza pandemic in the Australian army. J. Infect. Dis..

[21] Morens D.M., Taubenberger J.K., Fauci A.S. (2008). Predominant role of bacterial pneumonia as a cause of death in pandemic influenza: Implications for pandemic influenza preparedness. J. Infect. Dis..

[22] Barnes H.L. (1918). The prophylactic value of Leary’s vaccine. JAMA.

[23] Black-Milne J., Rogers K. (1919). Influenza broncho-pneumonia and pneumonia treated with the Army mixed vaccine. Lancet.

[24] Eyre J.W.H., Lowe E.C. (1919). Report upon the autumn influenza epidemic 1918 as it affected the NZEF in the United Kingdom. Lancet.

[25] Rosenow E.C. (1919). Prophylactic inoculation against respiratory infections during the present pandemic of influenza. JAMA.

[26] Medical Research Committee (1919). Studies of Influenza in Hospitals of the British Armies in France, 1918.

[27] Opie E.L., Blake F.G., Small J.C., Rivers T.M. (1921). Epidemic Respiratory Disease: The Pneumonias and Other Infections of the Respiratory Tract Accompanying Influenza and Measles.

[28] Chien Y.W., Klugman K.P., Morens D.M. (2010). Efficacy of whole-cell killed bacterial vaccines in preventing pneumonia and death during the 1918 influenza pandemic. J. Infect. Dis..

[29] Gras S., Kedzierski L., Valkenburg S.A., Laurie K., Liu Y.C., Denholm J.T., Richards M.J., Rimmelzwaan G.F., Kelso A., Doherty P.C. (2010). Cross-reactive CD8^+^ T-cell immunity between the pandemic H1N1-2009 and H1N1-1918 influenza A viruses. PNAS.

[30] Vidal S.M., Khakoo S.I., Biron C.A. (2011). Natural killer cell responses during viral infections: Flexibility and conditioning of innate immunity by experience. Curr. Opin. Virol..

[31] Damjanovic D., Small C.-L., Jeyananthan M., McCormick S., Xing Z. (2012). Immunopathology in influenza virus infection: Uncoupling the friend from foe. Clin. Immunol..

[32] Kedzierska K., Valkenburg S.A., Doherty P.C., Davenport M.P., Venturi V. (2012). Use it or lose it: Establishment and persistence of T cell memory. Front. Immunol..

[33] Shinya K., Okamura T., Sueta S., Kasai N., Tanaka M., Gunting T.E., Makino A., Eisfeld A.J., Kawaoka Y. (2011). Toll-like receptor pre-stimulation protects mice against lethal infection with highly pathogenic influenza viruses. Virol. J..

[34] Wlodarczyk M.F., Kraft A.R., Chen H.D., Kenney L.L., Selin L.K. (2013). Anti–IFN-γ and peptide-tolerization therapies inhibit acute lung injury induced by cross-reactive influenza a-specific memory T cells. J. Immunol..

[35] Kim J.H., Davis W.G., Sambhara S., Jacob J. (2012). Strategies to alleviate original antigenic sin responses to influenza viruses. Proc. Natl. Acad. Sci. USA.

[36] Nabel G.J., Fauci A.S. (2010). Induction of unnatural immunity: Prospects for a broadly protective universal influenza vaccine. Nat. Med..

[37] Peiris J., Hui K.P., Yen H.L. (2010). Host response to influenza virus: Protection versus immunopathology. Curr. Opin. Immunol..

[38] Rockman S., Schoof P., Greenberg M. (2011). Development and testing of the Australian pandemic influenza vaccine—A timely response. Micro. Australia..

